# Comparative analysis between STANDARD‐E Covi‐FERON ELISA with pre‐existing IFN‐γ release assays and determination of the optimum cutoff value for assessment of T‐Cell response to SARS‐CoV‐2

**DOI:** 10.1002/jcla.24882

**Published:** 2023-04-09

**Authors:** Jieun Jang, Kristin Widyasari, Sunjoo Kim

**Affiliations:** ^1^ Gyeongnam Center for Infectious Disease Control and Prevention Changwon 51154 South Korea; ^2^ Gyeongsang Institute of Health Sciences Gyeongsang National University Jinju 52727 South Korea; ^3^ Department of Laboratory Medicine Gyeongsang National University Changwon Hospital Changwon 51472 South Korea

**Keywords:** COVID‐19, cutoff value, interferon‐gamma, interferon‐gamma release assay, kappa‐index, T‐cell response

## Abstract

**Background:**

Interferon‐gamma (IFN‐γ) release assays (IGRAs) are useful for the assessment of the T‐cell response to severe acute respiratory syndrome‐coronavirus‐2 (SARS‐CoV‐2). We aimed to assess the performance of the newly developed IGRA ELISA test compared to the pre‐existing assays and to validate the cutoff value in real‐world conditions.

**Methods:**

We enrolled 219 participants and assessed agreement between STANDARD‐E Covi‐FERON ELISA with Quanti‐FERON SARS‐CoV‐2 (QFN SARS‐CoV‐2), as well as with T SPOT Discovery SARS‐CoV‐2 based on Cohen's kappa‐index. We further determined the optimal cutoff value for the Covi‐FERON ELISA according to the immune response to vaccinations or infections.

**Results:**

We found a moderate agreement between Covi‐FERON ELISA and QFN SARS‐CoV‐2 before vaccination (kappa‐index = 0.71), whereas a weak agreement after the first (kappa‐index = 0.40) and second vaccinations (kappa‐index = 0.46). However, the analysis between Covi‐FERON ELISA and T SPOT assay demonstrated a strong agreement (kappa‐index >0.7). The cut‐off value of the OS (original spike) marker was 0.759 IU/mL with a sensitivity of 96.3% and specificity of 78.7%, and that of the variant spike (VS) marker was 0.663 IU/mL with a sensitivity and specificity of 77.8% and 80.6%, respectively.

**Conclusion:**

The newly determined cut‐off value may provide an optimum value to minimize and prevent the occurrence of false‐negative or false‐positive during the assessment of T‐cell immune response using Covi‐FERON ELISA under real‐world conditions.

## Graphical abstract

The presence of IFN‐γ in the patient's blood samples can be an indicator of SARS‐CoV‐2 infection or the development of cellular immune response following the COVID‐19 vaccination. An IFN‐γ release assay (IGRA) enables the detection of IFN‐γ in the blood, and, therefore, plays an important role in the detection of SARS‐CoV‐2 or confirmation of the COVID‐19 vaccine's efficacy. Subsequently, an optimum cutoff value of the IGRA's markers is crucial to determine the accuracy of the IGRA test in discriminating the presence or absence of IFN‐γ in the blood samples.

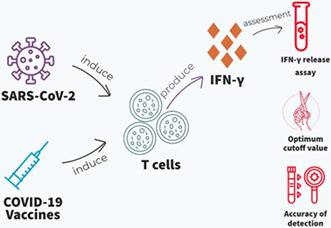

## 1 INTRODUCTION

Coronavirus Disease 2019 (COVID‐19) continues to be a global concern despite various efforts that have been made to control its spread. Numerous extensive studies of the immune response to COVID‐19 vaccination or to infections by SARS‐CoV‐2, which causes COVID‐19, have elucidated the significance of T‐cells in COVID‐19 immunity.^1^, ^2^, ^3^, ^4^, ^5^, ^6^ One method for assessing the T‐cell's response to SARS‐CoV‐2 is the interferon‐gamma (IFN‐γ) release assay (IGRA), which is a blood‐based test for diagnosing infectious diseases by measuring the IFN‐γ secreted by antigen‐specific T‐helper cells (Th cells) in blood samples.^7^ The IGRA test was introduced in 2001 for the assessment of the IFN‐γ released by T‐cells following stimulation by antigen‐specific to *Mycobacterium tuberculosis*.^8^ Following the COVID‐19 pandemic, the IGRA assays for SARS‐CoV‐2 were also developed. Currently, two types of IGRA tests are available for evaluation of the IFN‐γ released by T‐cells following the COVID‐19 vaccination or SARS‐CoV‐2 infection: Quanti‐FERON (QFN) SARS‐CoV‐2 (QIAGEN) and T SPOT Discovery SARS‐CoV‐2 kit (Oxford Immunotec).

The Quanti‐FERON (QFN) SARS‐CoV‐2 is a whole blood‐based assay that can detect IFN‐γ produced by CD4+ and CD8+ T‐cells in response to a SARS‐CoV‐2 peptide in the blood.^9^ This assay is based on the same platform as the Quanti‐FERON‐TB Gold plus, which is a well‐established diagnostic tool for latent tuberculosis infection.^10^ By the end of 2021, the Quanti‐FERON (QFN) SARS‐CoV‐2 has obtained a Conformite Europeenne (CE) mark and is deemed to meet EU safety, health, and environmental protection requirements; hence, it can be widely used to assess the T‐cell response to COVID‐19.^11^ Meanwhile, the T SPOT Discovery SARS‐CoV2 kit is a peripheral blood mononuclear cell (PBMCs)‐based assay that allows the detection and enumeration of the SARS‐CoV‐2 specific T‐cells.^12^ Both types of IGRA tests have been used to evaluate the significance and durability of the T‐cell response to SARS‐CoV‐2 infection or the COVID‐19 vaccine in several published studies.^13^, ^14^ Nevertheless, although the IGRA kit showed potential as a T‐cell evaluation tool, the sensitivity and accuracy of the IGRA assay remain an issue that requires further investigation.^15^, ^16^

The STANDARD‐E Covi‐FERON ELISA (RUO; SD BIOSENSOR), is a newly developed IGRA kit to evaluate the T‐cell mediated immune response to SARS‐CoV‐2 specific proteins in heparinized whole blood. Although this kit is currently for research use only, studies on the T‐cell response to SARS‐CoV‐2 using this kit demonstrated the potential of the STANDARD‐E Covi‐FERON ELISA as a useful tool for assessing T‐cell response against SARS‐CoV‐2.^17^, ^18^ Hence, evaluation of whether the results from Covi‐FERON ELISA are comparable to the results from pre‐existing IGRAs is necessary. Additionally, given that the sensitivity of the IGRA kit remains an issue, and that the sensitivity and specificity of the test are dependent on the cut‐off value, it is crucial to validate whether or not the manufacturer's provided cut‐off value is optimal in discriminating between positive or negative cases during the diagnostic test in the practical conditions. Therefore, in this study, we aimed to analyze the agreement between the newly developed IGRA kit, the STANDARD‐E Covi‐FERON ELISA, with two pre‐existing IGRA assays, Quanti‐FERON SARS‐CoV‐2, and T SPOT Discovery SARS‐CoV‐2 and determined the optimal cutoff value of STANDARD‐E Covi‐FERON ELISA according to the immune response that developed following SARS‐CoV‐2 infection and/or COVID‐19 vaccination under real‐world conditions. We also evaluated the performance of the STANDARD‐E Covi‐FERON ELISA when those newly determined cutoff values were applied.

## 2 MATERIALS AND METHODS

### 2.1 Study setting

This study was based on a longitudinal study design with 219 participants with a median age of 37. The enrolled participants comprised 41 COVID‐19 patients and 178 healthy volunteers confirmed by real‐time PCR tests who have fully received the COVID‐19 vaccines. The study period was from June 2021 to February 2022. People with a history of specific allergies, pregnant women, or someone receiving immunosuppressants were excluded from the study. All the participants provided written informed consent.

### 2.2 Sample collection

Blood samples were collected at two time points from COVID‐19 patients (before vaccination with at least 4 months after confirmed diagnosis and 4 weeks after vaccination with either BNT162b2 [Pfizer] or ChAdOx1‐S [AstraZeneca]); and at three‐time points from healthy volunteers (1 day before vaccination and 4 weeks following the first and second doses of ChAdOx1‐S or BNT162b2 vaccines).

### 2.3 Covi‐FERON ELISA assay

The STANDARD‐E Covi‐FERON ELISA hereinafter refers to as Covi‐FERON ELISA assay was performed according to the manufacturer's instruction. In brief, whole blood specimens were collected from the participants, and 1 mL was distributed into each Covi‐FERON tube (Nil tube [negative control], SARS‐CoV‐2 original spike protein tube [OS], SARS‐CoV‐2 variant spike protein tube [VS], and mitogen tube [positive control]). All tubes were incubated at 37°C for 24 h and then centrifuged at 2300 *g* for 15 min to harvest the plasma. The obtained plasma samples were subjected to ELISA to determine the amount of IFN‐γ. The predetermined cutoff value was ≥0.25 IU/mL in the manufacturer's instruction.

### 2.4 Quanti‐FERON SARS‐CoV‐2 assay

The Quanti‐FERON SARS‐CoV‐2 assay hereinafter refers to as QFN SARS‐CoV‐2 was performed according to the manufacturer's instructions. Briefly, whole blood specimens were collected from the participants, and 1 mL was distributed into each of the QFN SARS‐CoV‐2 tubes (Nil tube [negative control], AG1 tube contains CD4^+^ epitopes derived from the receptor binding domain (RBD) that detect IFN‐γ production by CD4^+^ [AG1]; the AG2 tube contains CD4^+^ and CD8^+^ epitopes from S1 and S2 subunits that detect IFN‐γ production by CD4^+^ and CD8^+^ [AG2], and mitogen tube [positive control]). All tubes were incubated at 37°C for 24 h and then centrifuged at 2300 *g* for 15 min to harvest the plasma. The obtained plasma samples were subjected to ELISA to determine the amount of IFN‐γ. The pre‐determined cutoff value was ≥0.20 IU/mL. The OS and VS markers in the Covi‐FERON ELISA and the AG1 and AG2 markers in the QFN SARS‐CoV‐2 were interpreted along with the other marker measurements from each kit to provide binary results for the presence or absence of the T‐cell immune response to SARS‐CoV‐2.

### 2.5 T SPOT Discovery SARS‐CoV‐2 assay

The T‐SPOT Discovery SARS‐CoV‐2 assay, hereafter referred to as the T‐SPOT assay, was performed according to the manufacturer's instructions. As described before,^19^ the peripheral blood mononuclear cells (PBMCs) were isolated from 5 mL of whole blood samples and washed to remove any sources of interfering background signals. Six wells were prepared for each sample: one nil control to identify non‐specific cell activation, three wells to assess the SARS‐CoV‐2‐specific antigens (panel 1 against SARS‐CoV‐2 spike protein, panel 3 against SARS‐CoV‐2 nucleocapsid protein, and panel 4 against SARS‐CoV‐2 membrane protein), one well to investigate cross‐reactivity with endemic strains of coronaviruses (panel 13), and one positive control, which was a mitogen solution containing phytohemagglutinin to confirm the functionality of PBMCs. Reactivity to panels 1, 3, and 4 indicated the response of T‐cells to SARS‐CoV‐2‐specific antigens, whereas reactivity to well 13 indicated cross‐reactivity with other coronaviruses. The predetermined cut‐off value was ≥8 SFCs/250,000 PBMCs. The results were defined as “invalid” when the spot‐forming cells (SFCs) of Nil control, are >10 spots or when the SFCs of positive control, are <20 spots. The results were defined as “reactive” when the SFCs in at least one of the three SARS‐CoV‐2 antigen wells (panels 1, 3, and 4) minus the SFCs of Nil control are ≥8 and defined as ‘non‐reactive’ when the SFCs in antigen wells minus SFCs of Nil control are <8.

### 2.6 Statistical analysis

We assessed the agreement between Covi‐FERON ELISA and QFN SARS‐CoV‐2 or between Covi‐FERON ELISA and T SPOT assay using Cohen's kappa‐index and corresponding 95% confidence interval (95% CI). We determined the optimal cutoff value of the Covi‐FERON ELISA markers to best discriminate the subjects with T‐cell immune response and without response by building the generalized mixed model and creating the receiver operating characteristic area under the curve (ROC‐AUC). The optimal cut‐off value for each marker was defined as the point that maximized the summation of the sensitivity and specificity values. The R packages, including “lmer,” and “pROC” were used to build the generalized mixed model, to draw the ROC‐AUC, and to determine the optimal cut‐off value of Covi‐FERON ELISA markers.

## 3 RESULTS

### 3.1 Agreement of Covi‐FERON ELISA and pre‐existing IGRA assays in detecting the IFN‐γ from the samples.

Results from our study demonstrated a moderate agreement (kappa‐index value [95% CI], 0.71 [0.57–0.86]) between Covi‐FERON ELISA and QFN SARS‐CoV‐2 for determination of the IFN‐γ concentration from the samples before administration of COVID‐19 vaccine. The agreement between Covi‐FERON ELISA and QFN SARS‐CoV‐2, however, decreased when the assessments were conducted for the samples obtained following the first and second vaccination with kappa‐index (95% CI) of 0.40 (0.27–0.54) and 0.46 (0.32–0.60), respectively (Table 1).

**TABLE 1:** Agreement between Covi‐FERON ELISA and Quanti‐FERON SARS‐CoV‐2 results according to vaccination status.

Vaccination status			Covi‐FERON ELISA		Kappa‐index (95% CI)
			Negative	Positive	
Before vaccination	Quanti‐FERON	Negative	100	13	0.71 (0.57–0.86)
		Positive	0	22	
First vaccination		Negative	43	49	0.40 (0.27–0.54)
		Positive	1	62	
Second vaccination		Negative	31	41	0.46 (0.32–0.60)
		Positive	3	126	

Agreement analysis of Covi‐FERON ELISA and T SPOT assay demonstrated a strong agreement between two IGRA test kits with kappa‐index values (95% CI) of 0.83 (0.71–0.94) and 0.81 (0.71–0.92) for samples obtained before vaccination and after the first COVID‐19 vaccination, respectively. Meanwhile, the agreement between Covi‐FERON ELISA and T SPOT assay after the second vaccination was slightly decreased but still considered as a moderate agreement, with a kappa value (95% CI) of 0.74 (0.61–0.87; Table 2).

**TABLE 2:** Agreement between Covi‐FERON ELISA and T‐SPOT assay results according to vaccination status.

Vaccination status			Covi‐FERON ELISA		Kappa‐index (95% CI)
			Negative	Positive	
Before vaccination	T‐SPOT	Negative	100	8	0.83 (0.71–0.94)
		Positive	0	26	
First vaccination		Negative	36	4	0.81 (0.71–0.92)
		Positive	7	94	
Second vaccination		Negative	25	5	0.74 (0.61–0.87)
		Positive	9	153	

### 3.2 Performance of the Covi‐FERON ELISA in detecting IFN‐γ using the newly determined cutoff value under real‐world conditions

Among three IGRAs used in this study, Covi‐FERON ELISA and QFN SARS‐CoV‐2 enable the detection and measurement of IFN‐γ as a manifestation of the T‐cell immune response to SARS‐CoV‐2. For both markers of Covi‐FERON ELISA (OS and VS), significantly higher IFN‐γ concentrations were detected in the COVID‐19‐positive group than one in the negative group (median of 2.49 IU/mL and 0.02 IU/mL, for OS marker, respectively, and median of 1.83 IU/mL and 0.02 IU/mL, for VS marker, respectively). Subsequently, the concentration of IFN‐γ was observed to be higher following the administration of the COVID‐19 vaccine than before vaccination, in both COVID‐19‐positive (median value: 5.35 IU/mL for OS and 1.87 IU/mL for VS) and negative groups (median value: 0.69 IU/mL for OS and 0.36 IU/mL for VS; Table 3). Similarly, analysis of IFN‐γ by QFN SARS‐CoV‐2 demonstrated a higher concentration of IFN‐γ in the COVID‐19‐positive group than the one in the negative group, for both AG1 and AG2 markers. Likewise, the median of IFN‐γ was elevated after the vaccine administration than that before vaccination.

**TABLE 3:** The concentration of IFN‐γ (IU/mL) was assessed by Covi‐FERON ELISA and Quanti‐FERON SARS‐CoV‐2 markers for infected and uninfected groups.

Markers	Group	Before vaccination	After vaccination
		Median (IQR)	Median (IQR)
Covi‐FERON ELISA OS	Infected	2.49 (1.77–9.66)	5.35 (1.78–7.80)
	Uninfected	0.02 (0.00–0.11)	0.69 (0.25–2.09)
Covi‐FERON ELISA VS	Infected	1.83 (0.90–7.88)	1.87 (0.69–4.04)
	Uninfected	0.02 (0.00–0.09)	0.36 (0.12–1.04)
Quanti‐FERON ELISA AG1	Infected	0.73 (0.33–3.71)	0.79 (0.20–1.49)
	Uninfected	0.00 (0.00–0.02)	0.23 (0.08–0.55)
Quanti‐FERON ELISA AG2	Infected	0.87 (0.49–3.86)	1.29 (0.55–3.26)
	Uninfected	0.00 (0.00–0.02)	0.36 (0.12–0.90)

Abbreviations: IQR, interquartile range; OS, original spike; VS, variant spike.

For the T SPOT assay, a higher number of spots forming cells (SFCs) was observed in the COVID‐19‐positive group than one in the negative group. Subsequently, observation of the SFCs among the groups according to the vaccination status demonstrated a substantial increase of SFCs in the COVID‐19‐negative group (median value: 0.0 before vaccination and 15.0 after vaccination), but only a slight elevation in the COVID‐19‐positive group (median value: 33.5 before vaccination and 36.0 after vaccination; Table 3).

**FIGURE 1:**
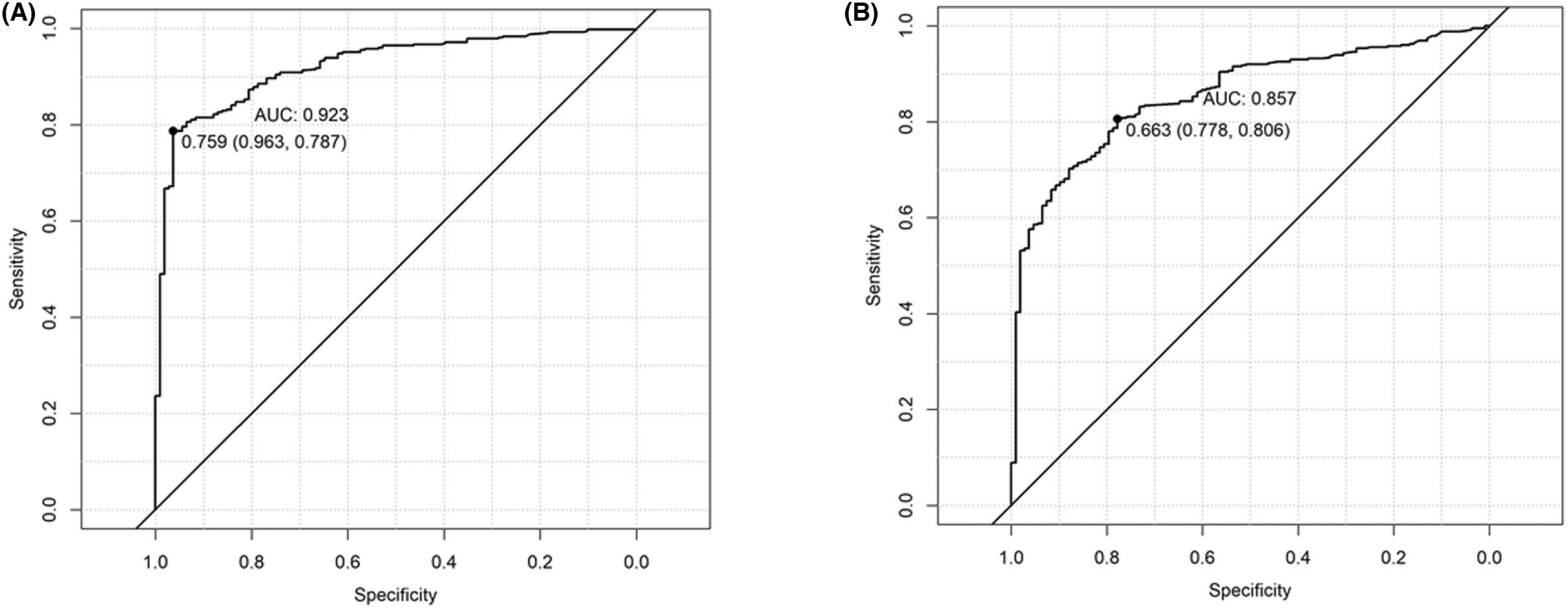
The receiver operating characteristic (ROC) curve of Covi‐FERON ELISA markers to discriminate individuals with and without a T‐cell immune response. (A) The optimal cutoff value of the Covi‐FERON ELISA original SARS‐CoV‐2‐spike protein (OS) marker is 0.759 IU/mL. (B) The optimal cutoff value of the Covi‐FERON ELISA variant SARS‐CoV‐2‐spike protein (VS) marker is 0.663 IU/mL.

We also determined the new cutoff value for Covi‐FERON ELISA's markers according to the immune response status of the participants (with the immune response defined by COVID‐19‐positive individuals or had received vaccination; without immune response defined by COVID‐19‐negative healthy individuals before vaccination). Our analysis demonstrated the optimal cut‐off value of the Covi‐FERON ELISA's OS marker as 0.759 IU/mL with a sensitivity and specificity of 98.3% and 78.7%, respectively under real‐world conditions (Figure 1A). Meanwhile, the cutoff value of the Covi‐FERON ELISA's VS marker was 0.663 IU/mL, with a sensitivity up to 77.8% and specificity of up to 80.6% (Figure 1B). These newly determined cutoff values of Covi‐FERON ELISA's OS and VS markers are almost three times higher than the suggested cutoff value by the manufacturer (0.25 IU/mL for both OS and VS markers).

## 4 DISCUSSION

The Covi‐FERON ELISA is an IGRA kit that enables the detection of IFN‐γ released by T‐cells as a response to SARS‐CoV‐2. The test comprised two markers that enabled the detection of T‐cell‐mediated immune response to SARS‐CoV‐2 Wuhan strain and alpha variant (B.1.1.7) specific spike proteins, known as the OS marker, and the one that enabled the detection of T‐cell response against a beta variant (B.1.351) and gamma variant (P.1) specific spike protein, known as the VS marker. A study on the performance of the Covi‐FERON ELISA reported that this IGRA kit is considered good and comparable with other commercial assays.^17^ However, given that Covi‐FERON ELISA is a newly developed IGRA kit, re‐evaluation of whether this IGRA kit has sufficient performance in real‐world conditions is crucial. For this purpose, we assessed the agreement of the Covi‐FERON ELISA with the pre‐existing/widely used IGRA assays and determined the most appropriate cutoff value of the Covi‐FERON ELISA's marker in real‐world conditions to avoid misinterpretation.

In this study, we performed an agreement analysis between the Covi‐FERON ELISA with pre‐existing IGRA assays (QFN SARS‐CoV‐2 and T SPOT) and found a weak to moderate agreement between Covi‐FERON ELISA and QFN SARS‐CoV‐2 and a strong concordance between Covi‐FERON ELISA and T SPOT assay for the evaluation of samples obtained before and after vaccination. The weaker agreement between Covi‐FERON ELISA and QFN SARS‐CoV‐2 occurs when we assessed samples collected after vaccination. The high discrepancy in the results was mainly due to samples being determined as reactive (positive) by Covi‐FERON ELISA but non‐reactive (negative) when being assessed by QFN SARS‐CoV‐2.

Further, we determined the optimal cutoff value for Covi‐FERON ELISA's markers according to the immune response that developed in real‐world conditions and demonstrated that the newly determined cutoff values for OS and VS markers are almost three times higher than the ones provided by the manufacturer. Hence, we assumed that the cutoff values which were pre‐determined by the manufacturer were too low, thus may cause false positives during the assessment after vaccination. The occurrence of false positives in the assessment likely leads to a high discrepancy between Covi‐FERON ELISA and QFN SARS‐CoV‐2. Together, our results suggest the importance of re‐assessment and re‐adjustment of the optimal cutoff value for the newly developed IGRA test kits in real‐world conditions to avoid any misinterpretation.

A raise of the cut‐off value usually leads to a test with high specificity but low sensitivity.^20^ Nevertheless, our study demonstrated that the newly determined cut‐off values give the Covi‐FERON ELISA a sensitivity as high as 96.3% and a specificity as high as 78.7% for the OS marker, and a sensitivity of 77.8%, a specificity of 80.6% for VS marker when being used for assessment in real‐world conditions. This result implies a considerably good performance of Covi‐FERON ELISA when the assessment is conducted by incorporating the new cutoff value for OS and VS markers.

The inability to find a gold standard for IGRA assessment has become a major limitation in our study. The determination of the optimal cutoff value for Covi‐FERON ELISA markers in our study was conducted by operationally defining the immune response status of participants. Immune response was defined as above mentioned: either from COVID‐19‐positive individuals or individuals who had received COVID‐19 vaccination. However, this operational definition may also be a limitation since the T‐cell immune response (IFN‐γ) may not yet be produced even after vaccination, especially in the individuals who only received a single dose of vaccine. Therefore, further study and subsequent efforts are needed to find a more accurate optimal cutoff value based on a more accurate gold standard for the assessment of the T‐cell response.

In conclusion, this study showed that the optimal cutoff value of markers in the Covi‐FERON ELISA, which measures IFN‐γ against SARS‐CoV‐2, should be adjusted somewhat higher than the value provided by the manufacturer. When the cutoff value of markers was adjusted, the performance of Covi‐FERON ELISA is considerably better than using the manufacturer's provided cutoff values. Given the potential of Covi‐FERON ELISA as a promising IGRA kit for measuring T‐cell response against SARS‐CoV‐2, additional efforts to find the appropriate threshold value of the Covi‐FERON ELISA markers under real‐world conditions remain necessary to minimize and avoid the occurrence of false positives during the assessment of T‐cell response to SARS‐CoV‐2 infection or COVID‐19 vaccination.

## AUTHOR CONTRIBUTIONS

Conceptualization: J. J. and S. K.; statistical analyses, writing results and methods sections: J. J.; writing–original draft preparation: K. W.; writing–review and editing: K. W. and S. K.; supervision and funding acquisition: S. K. All authors have read and agreed to the final version of the manuscript.

## FUNDING INFORMATION

This research was supported by the National Research Foundation (NRF) of Korea (NRF‐2021R1I1A3044483 and NRF‐2021M3E5E3080382). The funders had no role in the study design, data collection, interpretation, or decision to submit the manuscript for publication.

## CONFLICT OF INTEREST STATEMENT

No potential conflict of interest was reported by the authors.

## INSTITUTIONAL REVIEW BOARD STATEMENT

This study was approved by the institutional review board (IRB) of Gyeongsang National University Changwon Hospital (IRB No. 2021‐03‐020).

## Data Availability

Data from this study are available upon reasonable request.
